# Simultaneous detection of human norovirus GI, GII and SARS-CoV-2 by a quantitative one-step triplex RT-qPCR

**DOI:** 10.3389/fmicb.2023.1269275

**Published:** 2024-01-08

**Authors:** Hua-Wei Yi, Xian-Mo Wang, Xin Tan, Cai-Zhi Ding, Chang-Li Zhang, Jia-Hao Wu, Qi Li, Chen-Qi Xin, Wen Fan

**Affiliations:** ^1^The First Affiliated Hospital of Yangtze University, Jingzhou, Hubei, China; ^2^The First People’s Hospital of Jingzhou, Jingzhou, Hubei, China; ^3^Health Science Center of Yangtze University, Jingzhou, Hubei, China; ^4^The People’s Hospital of Songzi, Jingzhou, Hubei, China

**Keywords:** norovirus, SARS-CoV-2, nucleic acid detection, RT-qPCR, genotyping

## Abstract

**Background:**

There are many similarities in the clinical manifestations of human norovirus and SARS-CoV-2 infections, and nucleic acid detection is the gold standard for diagnosing both diseases. In order to expedite the identification of norovirus and SARS-CoV-2, a quantitative one-step triplex reverse transcription PCR (RT-qPCR) method was designed in this paper.

**Methods:**

A one-step triplex RT-qPCR assay was developed for simultaneous detection and differentiation of human norovirus GI (NoV-GI), GII (NoV-GII) and SARS-CoV-2 from fecal specimens.

**Results:**

The triplex RT-qPCR assay had high detection reproducibility (CV < 1%) and sensitivity. The lower limits of detection (LLOD95) of the triplex RT-qPCR assay for each target site were 128.5–172.8 copies/mL, and LLOD95 of the singleplex RT-qPCR assay were 110.3–142.0 copies/mL. Meanwhile, among the detection of clinical oropharyngeal swabs and fecal specimens, the results of the singleplex and triplex RT-qPCR assay showed high agreement.

**Conclusion:**

The triplex RT-qPCR assay for simultaneous detection of NoV-GI, NoV-GII and SARS-CoV-2 from fecal specimens has high clinical application value.

## 1 Introduction

Noroviruses (NoVs) are the primary causative agents of human non-bacterial acute gastroenteritis worldwide (Hall et al., 2013), and can be transmitted through various routes, including contaminated food, water, daily-life contact and other means (Ngazoa et al., 2008). According to genetic characteristics, NoVs are divided into 10 genogroups (GI-GX), the genogroups of human infection are GI, GII, GIV, GVIII and GIX (Chhabra et al., 2019). Among them, NoV-GI and NoV-GII are the main genogroups that cause acute gastroenteritis in humans (Rocha-Pereira et al., 2016). NoV infection is endemic throughout the year, with outbreaks peaking in March–April. The mainly infection targets are adults and school-age children, and often appearing in cluster outbreaks (Wang et al., 2018).

The incubation period after NoV infection is mostly 18–48 h, and the clinical manifestations include vomiting, abdominal cramps, fever, mucus in stool, watery diarrhoea, headache, chills and myalgia (De Graaf et al., 2016). These manifestations are highly reminiscent of those associated with coronavirus disease 2019 (COVID-19), particularly the current prevalence of SARS-CoV-2 omicron mutant strain, which reveal highly infectious and prone to fever, vomiting, diarrhea and other symptoms (Menni et al., 2022; Vihta et al., 2022). In some cases, it is difficult to distinguish NoV infection from COVID-19 only based on clinical symptoms. How to quickly and accurately distinguish between the two infections is crucial for treatment.

There are four main methods to detect NoV: electron microscopy, immunology, molecular biology and gene chip (Kapikian et al., 1972; Farkas et al., 2015; Kou et al., 2015). Among them, RT-qPCR is considered to be the most accurate and sensitive method for detection of NoV RNA in fecal specimens, and the method is also widely used in clinical detection (Pang and Lee, 2015). Besides, nucleic acid detection is the gold standard for the diagnosis of COVID-19 (Wang et al., 2021). Currently, oropharyngeal swabs, nasopharyngeal swabs and anal swabs have been widely used in mass SARS-CoV-2 screening, and the duration of nucleic acid-positive in anal swabs/feces is longer than in other samples (Jiang et al., 2020; Tang et al., 2020). Thus, the infection of NoV and SARS-CoV-2 can be quickly distinguished by identifying the pathogen in feces.

This study designed a triplex RT-qPCR assay, which can quickly distinguish NoV-GI, NoV-GII and SARS-CoV-2 from fecal specimens. To the authors’ knowledge, no multiplex RT-PCR assays have been reported for simultaneous detection of NoV and SARS-CoV-2.

## 2 Methods

### 2.1 Design of primers and probes

A triplex quantitative reverse transcription PCR (RT-qPCR) was designed for simultaneous detection of NoV-GI, NoV-GII and SARS-CoV-2. The primer and TaqMan probe sequences for NoV-GI and NoV-GII referred to previously published oligonucleotide primers (Liu et al., 2020). In order to cover as many NoV mutants as possible, three primers were designed to amplify NoV-GI and NoV-GII, and the TaqMan probes targeted ORF1-ORF2 junction region. In the nucleic acids detection of SARS-CoV-2, the N gene usually exhibits superior sensitivity and specificity compared with ORF1a/b target site (Zoka and Beko, 2020; Abbasi et al., 2022). Therefore, the N gene was used as the only target for SARS-CoV-2 detection. The primers and probes targeting the N gene of SARS-CoV-2 were consistent with the sequences provided by the National Microbiology Data Center (NMDC) of China, as described in https://nmdc.cn/nCoV. Primers, TaqMan probes and the target sites were described in Table 1. All the primers and probes were synthesized by Shanghai Sangon Biological Engineering Co., Ltd. (Shanghai, China) and purified by high performance liquid chromatography (HPLC).

**Table 1:** Primers/probes for the detection of NoV-GI, NoV-GII and SARS-CoV-2.

Target	Oligo	Primer/probe sequence (5′-3′)	Target site	Length (nt)	Amplicon size (bp)
NoV-GI	GI-F	GGAGATCGCRATCTCCTGCCCGA	ORF1-ORF2 junction	23	104
	GI-RA	CTCYGGTACCAGCTGGCC		18	
	GI-RB	CCTCYGGHACCAGCTGACC		19	
	Probe	HEX-CGTCCTTAGACGCCATCATCATT TAC-BHQ1		26	
NoV-GII	GII-FA	GTGGGATGGACTTTTACGTGCCAAG	ORF1-ORF2 junction	25	129
	GII-FB	GGTGGMATGGATTTTTACGTGCCCAG		26	
	GII-R	CGTCAYTCGACGCCATCTTCATTCAC		26	
	Probe	ROX-AGCCAGATTGCGATCGCC-BHQ2		18	
SARS-CoV-2	N-F	GGGGAACTTCTCCTGCTAGAAT	N gene	22	99
	N-R	CAGACATTTTGCTCTCAAGCTG		22	
	Probe	FAM-TTGCTGCTGCTTGACAGATT-BHQ1		20	

### 2.2 Extraction of viral nucleic acids

Nucleic acid extraction kit (magnetic beads method, Guangzhou Daan Gene Co., Ltd. China) and nucleic acid extractor were used to extract nucleic acids from clinical oropharyngeal swabs and fecal specimens. Viral RNA extraction was performed following the standard protocol (Chen et al., 2020). Oropharyngeal swab specimens can be directly used to extract nucleic acids, while fecal specimens need to be dissolved with an appropriate amount of physiological saline and shaken well, and 200 μL of supernatant after centrifugation was taken for nucleic acid extraction. The viral RNA was finally eluted with ~50 μL elution buffer and subsequently frozen in −80°C refrigerator.

### 2.3 Singleplex and triplex RT-qPCR assays

The RT-qPCR assays were performed using TaqMan probe one-step RT-qPCR 5G preMix (TOROIVD, China). 12.5 μL 2 × 5G qPCR buffer and 1.3 μL Enzyme Mix were added to the 25 μL reaction system. The final concentrations of primers in RT-qPCR assay were 400 nM, and the concentrations of TaqMan probes were 160 nM. Meanwhile, 5 μL template was added, and supplemented with ddH_2_O to 25 μL. In the triplex RT-qPCR assay, there are three pairs of amplification primers and probes, which can simultaneously detect viral RNA of NoV-GI, NoV-GII and SARS-CoV-2.

The reverse-transcription step was performed at 50°C for 15 min followed by incubation at 95°C for 5 min. Amplification included 45 cycles of denaturation at 95°C for 10 s, followed by annealing, extension and data acquisition at 60°C for 40 s on Applied Biosystems 7500 Fast Real-Time PCR system (Thermo fisher).

### 2.4 Preparation of viral RNA standards

The high concentration of SARS-CoV-2 RNA was used as the standard for N gene detection, and the virus RNA was extracted from an oropharyngeal swab sample of a SARS-CoV-2 strongly positive patient (cycle threshold (CT) value was 20.2). The concentration of the positive standard (1.1 × 10^6^ copies/mL) was determined by using a serial 10-fold dilution of the SARS-CoV-2 pseudovirus BDS-S1 (BDS Biological Technology Co., Ltd., Guangzhou, China; Cat. No. BDS-BW-118) as a quantitative standard curve for qPCR. The positive standards for NoV-GI and NoV-GII were extracted from RNA pseudovirus containing NoV gene fragments (Landes Medical Technology Co., Ltd., Hubei, China; Cat. No. FX10132303002). According to the results provided in the instructions, the concentrations of NoV-GI and NoV-GII standards were 2.2 × 10^6^ and 3.6 × 10^6^ copies/mL, respectively.

### 2.5 Standard curve of singleplex and triplex RT-qPCR assay

The prepared three viral RNA standards (NoV-GI, NoV-GII and SARS-CoV-2) were mixed equally, and serially diluted 10-fold with ddH_2_O to five dilutions (1×, 10×, 100×, 1,000×, 10,000×) for each target. The triplex and singleplex RT-qPCR assays were used to detect these viral RNA standard dilutions. Cycle threshold (Ct) values were plotted against log10 viral RNA concentrations to establish a standard curve, and linear regression analysis was performed for the three targets, allowing determination of the correlation coefficient (R^2^). The amplification efficiencies (E) of the reactions were calculated from the curves using the equation E = (10^(−1/slope)^ − 1) × 100% (Ni et al., 2021).

### 2.6 Reproducibility and specificity analysis of the triplex RT-qPCR assay

The three viral RNA standards (NoV-GI, NoV-GII and SARS-CoV-2) were prepared and mixed in equal proportions. The mixed standards were then diluted 10-fold and 1,000-fold with ddH_2_O as high and low concentration templates, respectively. The singleplex and triplex RT-qPCR assays were employed to detect the target sites of NoV-GI, NoV-GII and SARS-CoV-2 in 12 parallel experiments. To assess the reproducibility of RT-qPCR assays, coefficient of variation (CV) for Ct values were compared.

To investigate the detection specificity of the triplex RT-qPCR assay, we collected positive controls for nucleic acids detection of gastrointestinal and respiratory virus, such as rotavirus, adenovirus, influenza A virus, influenza B virus, respiratory syncytial virus and human rhinovirus. These controls were pseudoviruses that contain specific viral fragments and derived from commercialized detection kits (Guangzhou Daan Gene Co., Ltd. China). The specificity is characterized by the interference of other viruses on detection results of the triplex RT-qPCR assay.

### 2.7 Lower limit of detection (LLOD95)

The 95% lower limit of detection (LLOD95) and lower limit of quantification (LLOQ) of RT-qPCR were assessed by using the viral RNA positive standard as described above. LLOD95 was defined as the target concentration (dilution level) that could be detected with 95% probability. The positive standards of NoV-GI, NoV-GII and SARS-CoV-2 were diluted serially in sterile nuclease-free water. Eight concentrations ranging from 1: 400, 1: 2,000, 1: 4,000, 1: 10,000, 1: 20,000, 1: 40,000, 1: 80,000 and 1: 200,000 were tested in parallel by the target specific singleplex and triplex RT-qPCR assay to compare the sensitivity of detection. The RT-qPCR of each dilution was performed in quadruplicate, and the RT-qPCR was independently repeated four times. A total of 16 tests were performed for each concentration, and positive detection rates were counted at each dilution. LLOD is the measurand concentration that produces at least 95% positive replicates, and the LLOD95 was estimated by using logistic regression with target concentration (dilution level) as an explanatory variable and detection of the sample (pos/neg) as a response variable. The logistic regression curve is obtained by plotting:


$$y=\frac{1}{1+e^{-\beta 0-\beta 1x}}$$

Where x denotes log2 (concentration), and the two unknown parameters β_0_ and β_1_ are approximated by maximum likelihood (ML) estimation (Forootan et al., 2017; Persson et al., 2018).

The LLOQ was calculated based on the reported method (Forootan et al., 2017). This was done by calculating the SD for the responses of the replicate samples at the different concentrations, and the SD of the data was calculated from corresponding template concentration. The coefficient of variation (CV = 100 × SD/mean) for concentrations measured in replicates with qPCR were plotted to Log10 (measured template concentration).

### 2.8 Clinical evaluation

Clinical performance of the triplex RT-qPCR assay was evaluated by detecting different clinical specimens. Oropharyngeal swabs and fecal specimens of patients in Jingzhou First People’s Hospital (Jingzhou, China) from January to March 2023 were collected. A total of 351 oropharyngeal swabs were collected for detection the nucleic acid of SARS-CoV-2, and 32 positive specimens were detected by the commercial kit of Wuhan EasyDiagnosis Biomedicine Co., Ltd. (Hubei, China). One hundred and eighty-eight fecal specimens for NoV detection were collected, and 34 positive samples were detected by commercial kit of Landes Medical Technology Co., Ltd. (Hubei, China). The newly designed singleplex and triplex RT-qPCR assays were also used to detect these samples, and Bland–Altman agreement analysis was used to highlight the correlation between the Ct values of the singleplex and triplex assays using GraphPad Prism (GraphPad Software Inc., La Jolla, CA, United States).

## 3 Results

### 3.1 Standard curve of triplex RT-qPCR assay

**Figure 1:**
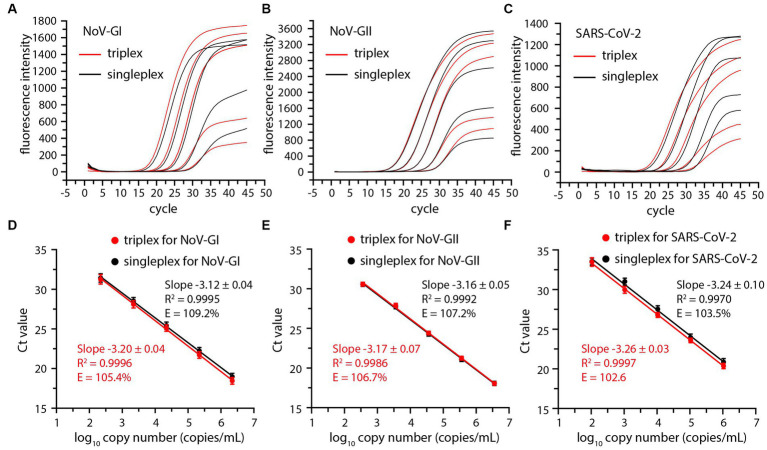
Comparative amplification plots and standard curves of the triplex and singleplex RT-qPCR using 10-fold serially diluted positive standards of the NoV-GI **(A,D)**, NoV-GII **(B,E)** and SARS-CoV-2 **(C,F)**.

In order to investigate the detection range of the triplex RT-qPCR, the viral RNA standards of NoV-GI, NoV-GII and SARS-CoV-2 were serially diluted 10-fold and serve as templates for detection. Thus, the amplification plots for triplex and singleplex RT-qPCR assays were obtained (Figures 1A–C).

Cycle threshold (Ct) values were plotted against log10 viral RNA concentrations to establish a standard curve, and there was no significant difference between triplex and singleplex assay curves (Figures 1D–F). The *R*^2^, slopes and amplification efficiency (E) of the standard curves were marked on Figure 1. The *R*^2^ of the standard curves were greater than 0.99. The E values of each detection target were within the range of 102–110%, and the results were in line with requirements (Broeders et al., 2014).

### 3.2 Reproducibility and specificity analysis of triplex RT-qPCR assay

To explore the reproducibility of RT-qPCR, we used singleplex and triplex RT-qPCR assays to detect nucleic acids of NoV and SARS-CoV-2 at high and low template concentrations, respectively. A total of 12 parallel experiments were conducted, the average Ct value and coefficient of variation (CV) of each target were shown in Table 2. The Ct values of the singleplex and triplex RT-qPCR assays did not differ significantly (*p* > 0.05). Meanwhile, the CV of Ct values were <1%. The results indicate that triplex RT-qPCR assay has high repeatability and is suitable for clinical detection.

**Table 2:** Reproducibility analysis of the singleplex and triplex RT-qPCR assay.

		Singleplex RT-qPCR			Triplex RT-qPCR		
	Template Conc	Average Ct	SD	CV%	Average Ct	SD	CV%
NoV-GI	High	22.30	0.21	0.94	22.01	0.10	0.45
	Low	28.52	0.05	0.17	28.24	0.06	0.21
NoV-GII	High	20.84	0.09	0.43	20.64	0.07	0.34
	Low	27.33	0.11	0.41	27.06	0.12	0.46
SARS-CoV-2	High	21.94	0.18	0.84	21.69	0.07	0.34
	Low	30.00	0.09	0.31	29.61	0.15	0.51

The triplex and singleplex RT-qPCR assays were tested on nucleic acids extracted from other gastroenteritis and respiratory viruses, including rotavirus, adenovirus, influenza A virus, influenza B virus, respiratory syncytial virus and human rhinovirus. The nucleic acids of these viruses were used as templates and then RT-qPCR experiments were performed. Neither assay found non-specific amplification signals for any target nucleic acids from these tested organisms, suggesting that the assays have good specificity.

### 3.3 Sensitivity analysis of triplex RT-qPCR assay

**Figure 2:**
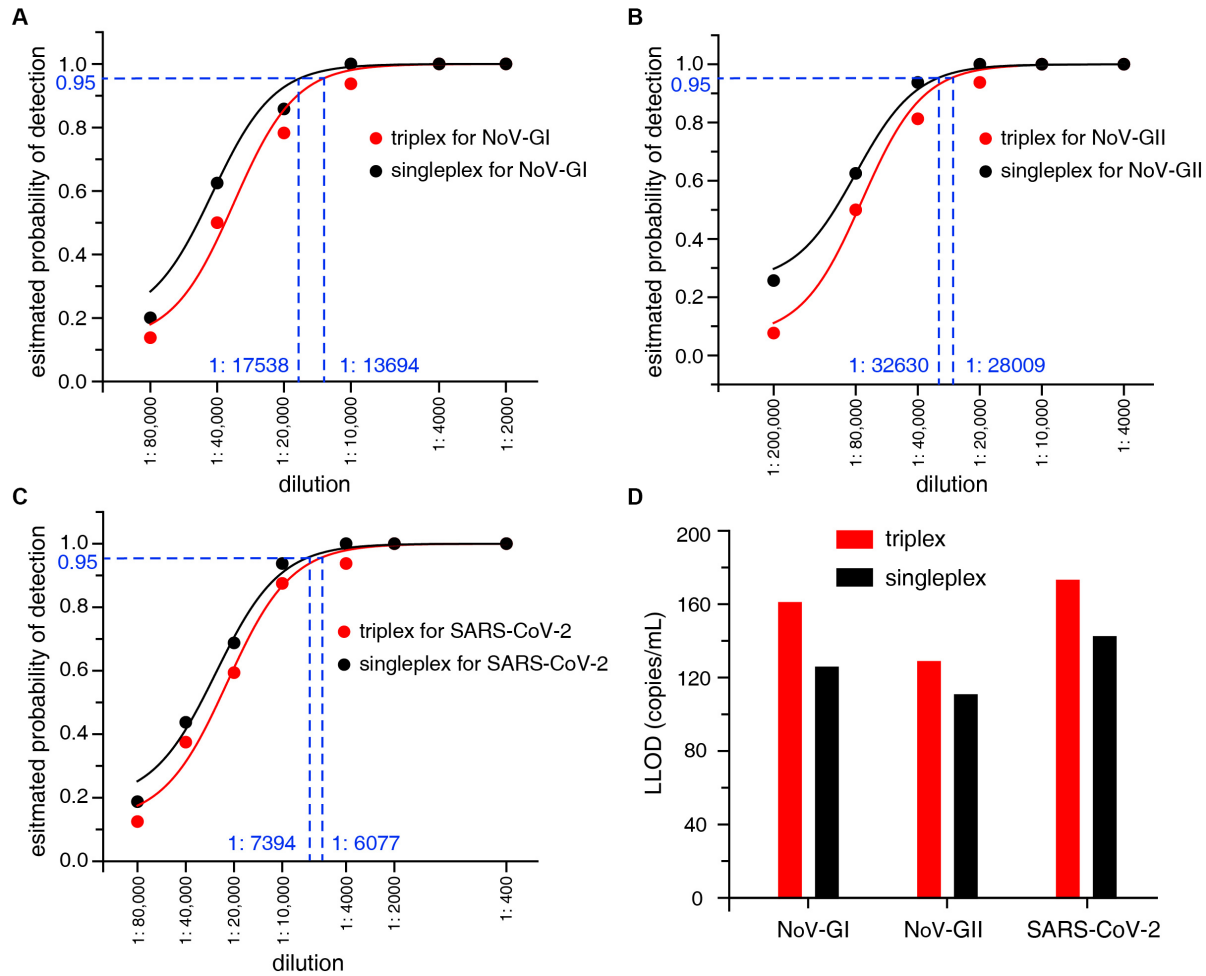
**(A–C)** LLOD95 was estimated by fitting a logistic regression model with detection results. Each dot indicates an actual detection rate at each dilution. The dashed line in the figure indicates the dilution ratio corresponding to a 95% probability. **(D)** Lower limit of detection (LLOD) comparison of the triplex and singleplex RT-qPCR for NoV-GI, NoV-GII and SARS-CoV-2.

The LLOD95 was utilized to evaluate the sensitivity of the RT-qPCR assays. The viral nucleic acid standards were serially diluted, and then the positive detection rates of three targets NoV-GI, NoV-GII and SARS-CoV-2 at each dilution were detected, as shown in Figures 2A–C. LLOD95 was estimated by logistic regression analysis as previously described (Persson et al., 2018). The LLOD95 results of triplex and singleplex RT-qPCR were shown in Figure 2D.

For the singleplex RT-qPCR assay, the LLOD was calculated to be 125.4 copies/mL for NoV-GI, 110.3 copies/mL for NoV-GII, and 142.0 copies/mL for SARS-CoV-2 (Figure 2D). For the triplex RT-qPCR assay, the LLOD was calculated to be 160.7 copies/mL for NoV-GI, 128.5 copies/mL for NoV-GII, and 172.8 copies/mL for SARS-CoV-2. The LLOD values of triplex assay were slightly lower than that of the singleplex. Additionally, there was no remarkable difference in LLOD values between them. This result indicates that the triplex and singleplex assays have the semblable sensitivity. In addition, lower limit of quantification (LLOQ) can also be estimated from the replicate standard curves (Forootan et al., 2017). The LLOQ of singleplex and triplex RT-qPCR assay for each target site were 309.7–393.6 copies/mL and 378.4–494.3 copies/mL, respectively (Supplementary Figure S1), and there was no significant difference in LLOQ between the two RT-qPCR assays.

### 3.4 Clinical evaluation

A total of 351 oropharyngeal swabs for detection of SARS-CoV-2 nucleic acid, and 188 fecal specimens for NoV detection were collected. Meanwhile, the nucleic acids of these samples were extracted using the magnetic beads method. The newly designed singleplex and triplex RT-qPCR detection assays were used to assess these samples. A total of 32 positive samples of SARS-CoV-2 and 34 positive samples of NoV were detected. The number of positive samples was consistent with the commercial detection kit. Interestingly, all 34 positive specimens of NoV were NoV-GII genogroup, which maybe result from the fact that NoV-GII is the most predominant NoV genogroup that causes human diseases in China (Kobayashi et al., 2016; Yu et al., 2022). In addition, one case positive for SARS-CoV-2 was detected in 34 NoV-positive fecal specimens.

**Figure 3:**
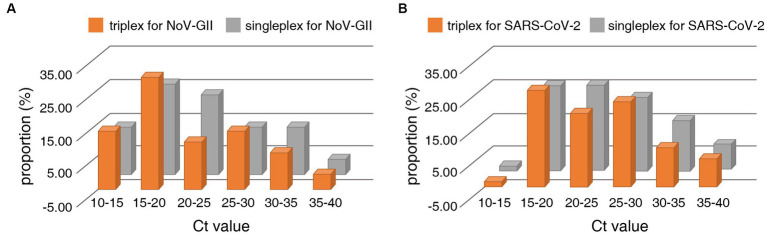
Distribution frequencies of Ct values for positive clinical samples analysed for detection of the NoV-II **(A)** and the N gene of SARS-CoV-2 **(B)** using the triplex and singleplex RT-qPCR.

**Figure 4:**
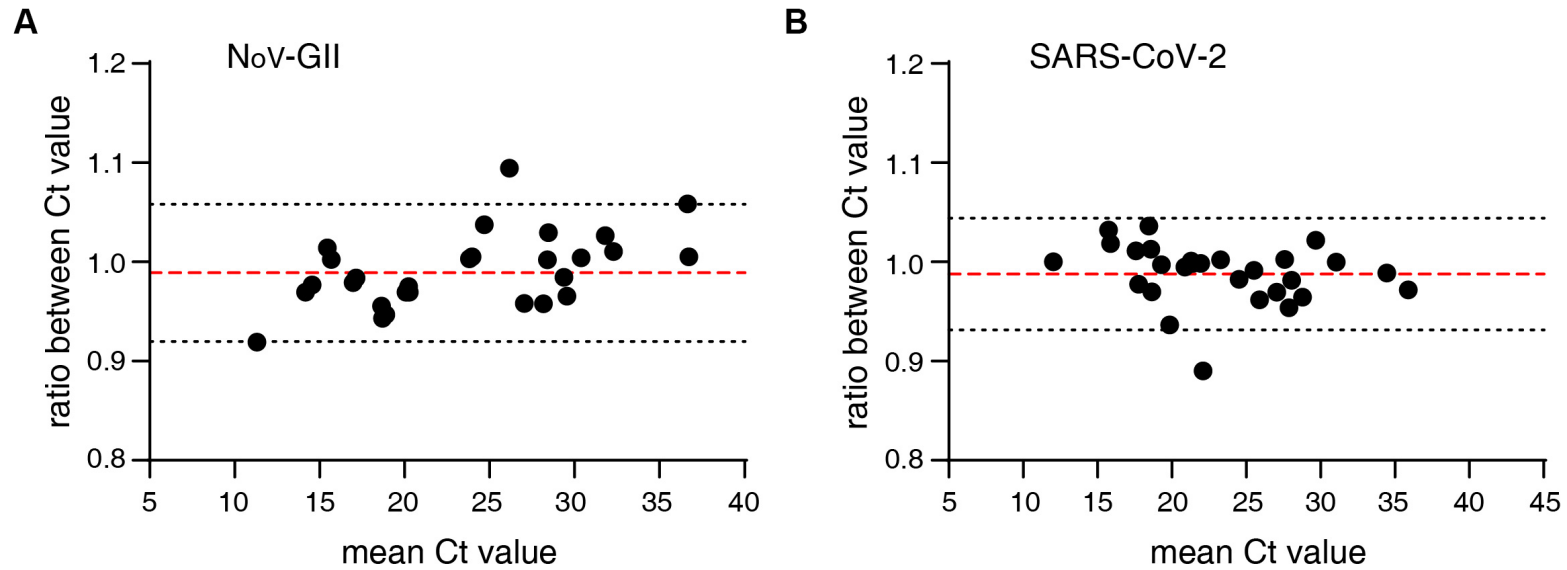
Bland–Altman agreement analysis of clinical specimens for the triplex and singleplex RT-qPCR assays. **(A)** Agreement analysis plot for the Ct value of NoV-GII. The mean of Ct ratio was 0.9890 (red dotted line), and the 95% limits of agreement was from 0.9197 to 1.058 (black dotted line). **(B)** Agreement analysis plot for the Ct value of SARS-CoV-2. The mean of Ct ratio was 0.9879 (red dotted line), and the 95% limits of agreement was from 0.9314 to 1.044 (black dotted line).

The distribution frequencies of Ct values for NoV-GII and SARS-CoV-2 of the clinical samples were displayed in Figure 3. For triplex and singleplex RT-qPCR, there was no significant difference in the proportion of each Ct value range. The Bland–Altman agreement analysis was used to highlight the correlation between the Ct values, and the agreement analysis plot was shown in Figure 4. 91.2% (31/34) of the positive points for the NoV-GII were within the 95% limits of agreement (LoA), and 96.8% (31/32) of the positive points for the SARS-CoV-2 were between the 95% LoA. The mean of Ct ratio for NoV-GII and SARS-CoV-2 was close to 1. This result indicates that the triplex and singleplex RT-qPCR assays have great consistency for detecting clinical specimens.

## 4 Discussion

A rapid triplex RT-qPCR assay for simultaneous detection and differentiation of NoV-GI, NoV-GII and SARS-CoV-2 was developed. Infection with NoV and SARS-CoV-2 present many similar symptoms, nausea vomiting and diarrhea accounted for approximately 20% of patients infected with the Delta and Omicron strains (Menni et al., 2022; Vihta et al., 2022). The triplex RT-qPCR designed in this study could detect the viral nucleic acid in fecal specimens, enabling to quickly distinguish NoV from SARS-CoV-2, as well as further differentiate between NoV-GI and NoV-GII. In the field of NoV and SARS-CoV-2 detection methodology, many multiple-qPCR methods have been established, and these methods have good sensitivity and specificity. For example, simultaneous detection of SARS-CoV-2, influenza A/B and respiratory syncytial viruses (RSV) (Domnich et al., 2023), detection of SARS-CoV-2 and influenza A/B (Ni et al., 2021), detection of NoV and Rotavirus A (Dung et al., 2013) and so on. However, to the authors’ knowledge, no multiplex RT-PCR assays have been reported for simultaneous detection of NoV and SARS-CoV-2.

In order to explore the detection capabilities of the newly designed triplex assay, the standard curves, reproducibility, lower limit of detection were compared with the singleplex RT-qPCR, and no significant differences were observed between those two assays. Meanwhile, the distribution frequencies of Ct values for the singleplex and triplex RT-qPCR assays displayed similarity, with highly consistent Ct values observed in the positive results. In addition, we also compared the triplex RT-qPCR assay with the commercial kits. Both singleplex and triplex RT-qPCR assays shown consistent results with commercial detection kits on clinical specimens, with a 100% coincidence rate of negative and positive. The LLOD95 determined by the triplex RT-qPCR assay for each target sites were 128.5–172.8 copies/mL, which was consistent with the reported results of RT-qPCR (Persson et al., 2018). Meanwhile, the LLOD was superior to the results provided by commercial kits (200 copies/mL). In this research, multiplex qPCR based on TaqMan probes was used to detect multiple target sites simultaneously. Real-time PCR melting curve analysis can also detect multiple sites and is also suitable for the simultaneous detection of NoV-GI, NoV-GII, and SARS-CoV-2. However, this method performs non-specific detection on amplified dsDNA with high false positives.

A limitation of this study was that only N gene was utilized for detecting SARS-CoV-2, lacking other detection sites such as ORF1a/b. In clinical detection of SARS-CoV-2, N gene usually has higher sensitivity and specificity than other sites (Zoka and Beko, 2020; Abbasi et al., 2022), and can also be used as a target site for various mutant strains of SARS-CoV-2. Besides, it is very common to detect pathogens through only one target site, such as nucleic acid detection of hepatitis B virus (HBV) and hepatitis C virus (HCV) (Candotti et al., 2004; Lall et al., 2019). In addition, there might be discrepancies between fecal specimens and oropharyngeal swabs in detecting nucleic acid of SARS-CoV-2. However, the vast majority of cases exhibit concordance between anal swabs/feces and oropharyngeal swabs, with a longer duration of nucleic acid-positive in anal swabs/feces compares to other samples (Jiang et al., 2020; Tang et al., 2020). Certainly, in many cases, there are significant differences in clinical symptoms between SARS-CoV-2 and NoV infections, which limits the widespread application of the triplex RT-qPCR assay.

In conclusion, the triplex RT-qPCR assay established in this paper plays an important role in the diagnosis and treatment of diseases that enable simultaneous detection of NoV-GI, NoV-GII and SARS-CoV-2 in fecal specimens, during the global spread of NoV and SARS-CoV-2.

## Data availability statement

The original contributions presented in the study are included in the article/Supplementary material, further inquiries can be directed to the corresponding authors.

## Ethics statement

The studies involving humans were approved by ethics committee of the First People’s Hospital of Jingzhou (no. KY202375). The studies were conducted in accordance with the local legislation and institutional requirements. The participants provided their written informed consent to participate in this study.

## Author contributions

H-WY: Conceptualization, Data curation, Funding acquisition, Writing – original draft, Writing – review & editing. X-MW: Conceptualization, Methodology, Project administration, Writing – review & editing. XT: Conceptualization, Formal analysis, Methodology, Writing – original draft. C-ZD: Resources, Methodology, Validation, Writing – review & editing. C-LZ: Resources, Writing – review & editing. J-HW: Investigation, Formal analysis, Methodology, Writing – review & editing. QL: Data curation, Investigation, Validation, Writing – review & editing. C-QX: Conceptualization, Data curation, Formal analysis, Resources, Writing – original draft, Writing – review & editing. WF: Conceptualization, Project administration, Supervision, Writing – original draft, Writing – review & editing.

## Supplementary material

SUPPLEMENTARY FIGURE S1 LLOQ was determined by estimating %CV and comparing concentrations measured in replicates with qPCR (Forootan et al., 2017). **(A-C)** Coefficient of variation (CV = 100 × SD/mean) for concentrations measured in replicates with qPCR were plotted to Log10 (measured template concentration). Horizontal blue dashed line indicates CV = 35% and vertical blue dashed line indicates the lowest concentration of samples with a CV below 35%. **(D)** LLOQ results of the triplex and singleplex assays. LLOQ results can be obtained based on the template concentration corresponding to 35% CV values.
