# Supplementary figures and images for: Simultaneous detection of human norovirus GI, GII and SARS-CoV-2 by a quantitative one-step triplex RT-qPCR
Source: Front Microbiol. 2024 Jan 8;14:1269275. doi: 10.3389/fmicb.2023.1269275 (PMC10800780; doi:10.3389/fmicb.2023.1269275)

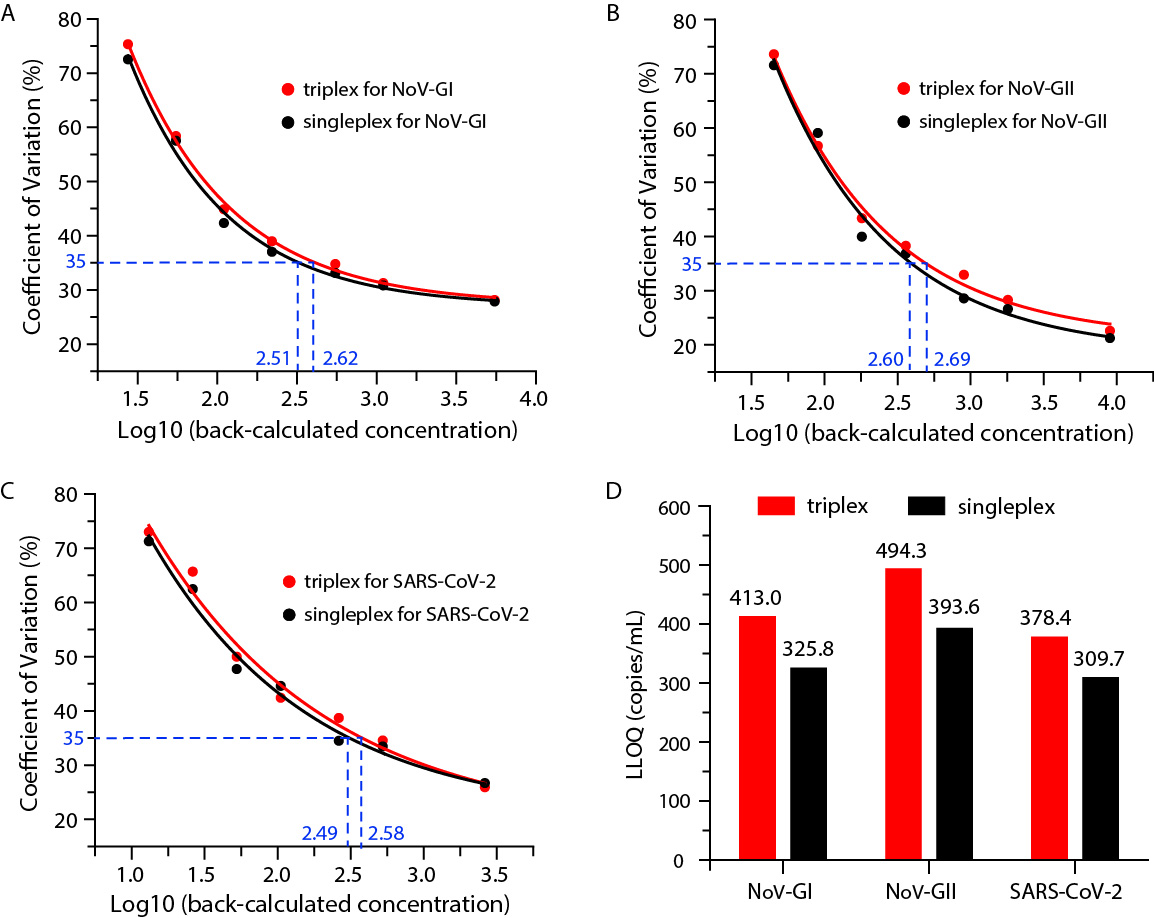

Supplement: SUPPLEMENTARY FIGURE S1 — LLOQ was determined by estimating %CV and comparing concentrations measured in replicates with qPCR (Forootan et al., 2017). (A-C) Coefficient of variation (CV = 100 × SD/mean) for concentrations measured in replicates with qPCR were plotted to Log10 (measured template concentration). Horizontal blue dashed line indicates CV = 35% and vertical blue dashed line indicates the lowest concentration of samples with a CV below 35%. (D) LLOQ results of the triplex and singleplex assays. LLOQ results can be obtained based on the template concentration corresponding to 35% CV values. [file Image_1.JPEG]
